# Genetic Predictors of Neurocognitive Outcomes in Survivors of Pediatric Brain Tumors

**DOI:** 10.21203/rs.3.rs-3225952/v1

**Published:** 2023-08-07

**Authors:** Sydney T. Grob, Kristen R. Miller, Bridget Sanford, Andrew M. Donson, Kenneth Jones, Andrea M. Griesinger, Vladimir Amani, Nicholas K. Foreman, Arthur Liu, Michael Handler, Todd C. Hankinson, Sarah Milgrom, Jean Mulcahy Levy

**Affiliations:** University of Colorado School of Medicine; University of Colorado School of Medicine; University of Colorado School of Medicine; University of Colorado School of Medicine; University of Colorado School of Medicine; University of Colorado School of Medicine; University of Colorado School of Medicine; University of Colorado School of Medicine; Children’s Hospital Colorado; Children’s Hospital Colorado; Children’s Hospital Colorado; Children’s Hospital Colorado; University of Colorado School of Medicine

**Keywords:** Pediatric brain tumor, neurocognitive outcome, single nucleotide polymorphism (SNP)

## Abstract

**Purpose:**

Neurocognitive deficits are common in pediatric brain tumor survivors. The use of single nucleotide polymorphism (SNP) analysis in DNA repair genes may identify children treated with radiation therapy for brain tumors at increased risk for treatment toxicity and adverse neurocognitive outcomes.

**Methods:**

The Human 660W-Quad v1.0 DNA BeadChip analysis (Illumina) was used to evaluate 1048 SNPs from 59 DNA repair genes in 46 subjects. IQ testing was measured by the Wechsler Intelligence Scale for Children. Linear regression was used to identify the 10 SNPs with the strongest association with IQ scores while adjusting for radiation type.

**Results:**

The low vs high IQ patient cohorts were well matched for time from first treatment to most recent IQ, first treatment age, gender, and treatments received. 5 SNPs on 3 different genes (CYP29, XRCC1, and BRCA1) and on 3 different chromosomes (10, 19, and 17) had the strongest association with most recent IQ score that was not modified by radiation type. Furthermore, 5 SNPs on 4 different genes (WRN, NR3C1, ERCC4, RAD51L1) on 4 different chromosomes (8, 5, 16, 14) had the strongest association with change in IQ independent of radiation type, first IQ, and years between IQ measures.

**Conclusions:**

SNP polymorphisms offer potential to predict adverse neurocognitive outcomes in pediatric brain tumor survivors. Our results require validation in a larger patient cohort. Improving the ability to identify children at risk of treatment related neurocognitive deficits could allow for better treatment stratification and early cognitive interventions.

## Introduction

There are approximately 2200 children diagnosed with brain tumors each year. As the overall survival of these children has improved from 57–74% in the interval from 1975 to 2002 [1]with 5-year survival reaching 75.5% in the time interval between 2013–2019[2] there is increasing concern over the neurocognitive deficits that accompany treatment for brain tumors. In addition to treatment related risk factors such as radiation dose [3]and surgery [4], several clinical risk factors for neurocognitive deficits have been identified. Some of these include gender [5, 6], younger age at diagnosis [3, 7], and children with hydrocephalus at presentation [8]. More defined biologic markers will make it possible to better predict patients at risk of long-term adverse neurocognitive effects following radiation therapy. This information could allow for better treatment stratification and early cognitive intervention in at-risk individuals.

There has been interest in examining the genetic factors that underlie normal tissue sensitivity to therapy. Numerous groups have examined genetic polymorphisms and radiation toxicity in adults with malignancies of the breast, prostate, head and neck, cervix, endometrium, and lung [9]. One study investigated the potential association between cognitive outcomes and single nucleotide polymorphisms (SNPs) in catechol-o-methyl transferase (*COMT*), brain-derived neurotrophic factor *(BDNF)*, and dystrobrevin-binding protein 1 *(DTNBP)*. These genes are associated with memory and daily functioning, and all genes are implicated in neurological impairment in adult tumor patients [10]. Other SNPs in the DIO1 gene, associated with control of thyroid hormone metabolism, were found to have a significant prognostic value in adult glioblastoma patients [11]. Most recently, a study found SNP polymorphisms in genes associated with aging, inflammation, dopamine, myelin cell cycle regulation, and DNA repair may be associated with neurocognitive outcomes in adult CNS tumor patients treated with radiation and chemotherapy [12].

There is a paucity of similar studies in children. In pediatric leukemia, certain SNPs, such as *UGT2B17*, have been correlated with treatment toxicity [13], neurocognitive outcomes [14, 15], and overall mortality. A deletion polymorphism in *UGT2B17* is thought to suppress tumor growth, which could contribute to an overall greater prognosis and reduced chance of relapse [16]. Polymorphisms in the ACYP2 [17] and SOD [18] genes have also been associated with differences in cisplatin-induces ototoxicity. These studies focused primarily on SNPs in genes involved in folate metabolism, drug detoxification DNA repair genes [19].

Evidence regarding the use of genetic profiling in the treatment of brain tumor patients is limited. Previous studies have utilized SNP analysis of the primary tumor to help determine treatment response, but there is little data predicting treatment toxicity. SNP analysis of Glutathione S-transferase [GST] has been studied in relation to neurocognitive toxicity. GST is an enzyme that catalyzes glutathione conjugation of alkylating agents, platinum compounds, and free radicals produced by radiation. A SNP analysis of GST in medulloblastoma patients found that the presence of a null genotype was associated with a significant decline in IQ after treatment compared to patients with a no null genotype [20]. Another study found that *GTSP1 105 AG/GG* genotypes were much more likely to experience radiation induced hearing loss. Furthermore, the G allele in combination with high dose radiation was associated with greater risk of treatment-induced toxicity overall [21].

A study of participants in the Childhood Cancer Survivor Study (CCSS) evaluated GST and other antioxidant enzyme SNPs to determine if they were associated with neuropsychological impairment. On a Brief Symptoms Inventory-18 questionnaire, patients with a GST null genotype reported increased anxiety, depression, and global distress compared to patients with a non-null genotype. But while the CCSS Neurocognitive Questionnaire found poorer functioning in task efficiency and memory by self-report when patients treated for medulloblastoma were compared to sibling controls, there was no difference between genotypes associated with this select set of antioxidant enzymes [22]. A study looking at the association between *COMT* polymorphisms, coding for an enzyme used in the metabolism related to control of dopamine levels within the prefrontal cortex and working memory in pediatric brain tumor survivors found that patients with the Met/Val polymorphism variant had a greater working memory performance [23].

More recent research has investigated the association between the three most common polymorphisms in Vitamin D located within the Bsm-1, Fok-1, and Taq-1 regions of the receptor in pediatric brain tumors [24]. The vitamin D receptor binds calcitriol which is involved in several cell processes including cell proliferation, apoptosis, tumorigenesis, cell invasion, inflammatory response [25]. This study found that the association between polymorphisms in the Vitamin D receptor and cancer development was insignificant, but no research was done correlating identified polymorphisms in Vitamin D to response to treatment [24]. Additional studies look to identify how SNPs may be used in the diagnostics and prognosis of brain tumors [26], but do not correlate these SNPs to toxicity outcomes.

Finally, a study looked to evaluate p53 Arg72Pro polymorphism as an early detector of tumor progression in pediatric astrocytoma and found that having the Arg/Arg72 variant can be used to predict early tumor growth in partially resected astrocytomas. This study went further to suggest that this polymorphism could be used to inform and predict individual response to therapy [27].

There is mounting evidence of a link between the ability to repair DNA damage and not only the development of cancer, but also the response to therapy [28]. This is important for pediatric brain tumor patients as standard of care therapy involves DNA damaging agents such as radiation and chemotherapy. We hypothesized that somatic genetic variants in DNA repair genes may be associated with lower IQ scores in children treated for brain tumors. We genotyped 46 children who had been treated for a pediatric brain tumor and assessed whether SNP array profiles were associated with differences in IQ scores.

## Methods and Materials

### Patient characteristics

Eligible patients had been previously treated for a brain tumor at Children’s Hospital Colorado and the University of Colorado Denver. Treatment included any combination of surgery, chemotherapy, and radiation therapy. We excluded children with known neurocognitive deficits prior to the initial diagnosis of the brain tumor. All children underwent IQ testing with the Wechsler Intelligence Scale for Children (WISC) as part of routine clinical follow-up. Our Institutional Review Board approved this study (COMIRB 08-0985).

### Laboratory Methods

DNA was extracted from patient blood samples using the Qiagen DNAeasy kit, per kit instructions. The DNA was analyzed using a Human 660W-Quad v1.0 DNA analysis BeadChip (Illumina) per the Infinium HD assay protocol as previously published [28]. Data output identified alleles by A and B designations, which were then converted to corresponding nucleotides after statistical analysis for further comparison.

### Statistical analysis

If normally distributed, continuous variables were summarized with mean and standard deviation (SD), and a two-sample t-test was used to compare across IQ groups (< = 90 versus > 90). Dichotomizing patients at an IQ of 90 was chosen as it’s the low end of the Wechsler Intelligence Scale for Children (WISC-V) IQ classification for “average” IQ [29]. If not normally distributed, continuous variables were summarized with median and interquartile range (IQR), and a Wilcoxon rank sum test was used to compare across IQ groups. Categorical variables were summarized with frequency and percentage, and comparisons were performed with a Chi-square or Fisher’s exact test.

#### Data Cleaning:

There were 47 patients in the original dataset. This contained serial IQ test results and basic clinical information like age, gender, diagnosis, radiation, and treatment details. One patient was unable to be linked to the SNP dataset so was removed from the final analysis. There were originally 732 unique SNPS whereby each SNP was categorized into AA versus other (AB, BB, NC), but 118 SNPS only had either all AA or all other so were removed from the final analysis leaving 614 for analysis.

#### Most recent IQ outcome:

For each of the 614 SNPs, a series of linear regression models were fit. For each SNP a model with the outcome of most recent IQ, predictors of allele (AA vs. others) and radiation type (CSI vs. focal), and an interaction between allele and radiation type was fit. If the interaction term was significant (p < 0.05), then that model was reported. If not, then the interaction was removed.

#### Change in IQ outcome:

only 24 patients who had at least 2 serial IQ measurements were used for analysis. Forty-nine SNPs had to be removed because they only had a single allele in this sub-population. Using the resulting 565 SNPs, the same series of linear regression models were fit, except each model used the change in IQ (most recent - next recent) as the outcome, and predictors of allele, radiation type, next recent IQ score, and the time between the two IQ measurements. The same decisions were made with the interactions between allele and radiation type.

It was decided a priori that the results would be ranked by the p-value of the allele term (or allele*radiation term, when applicable), and the most significant 10 SNPs would be reported for each outcome (most recent and change in IQ). Analysis was done in R version 4.2.1, and the significance level was set at 0.05.

## Results

Forty-six patients were enrolled in the study and had blood samples obtained for SNP genotyping and at least one IQ test completed. For descriptive purposes, the most recent IQ scores were dichotomized at 90 with 26 (56%) subjects having an IQ less than 90 and 20 (43%) subjects having an IQ greater than 90. The low vs high IQ patient cohorts were well matched for time from first treatment to most recent IQ test (median (IQR): 5.1 (2.7–7.6) vs 3.9 (2.7–4.6) years; p = 0.13), age at first treatment (6.2 (4.3–8.5) vs 6.2 (4-11.2) years; p = 0.71), and sex distribution (65% vs 70% male; p = 0.99). The cohorts differed non-significantly in their primary tumor diagnosis with 54% of patients in the low IQ being diagnosed with medulloblastoma in comparison to ependymoma (25%) and other (25%) being the most common diagnoses in the high IQ group. The primary tumor location for patients in both the low and high IQ groups was the posterior fossa (58% and 45%, respectively); although, the two groups’ tumor locations were significantly different (p = 0.02). The higher IQ group’s second most common location was the suprasellar/hypothalamic (25%) followed by the parietal (10%) and pineal (10%) regions which differed from the low IQ group where the second most common location was the thalamus (15%) and suprasellar/hypothalamic (12%). The groups did not differ in terms of proportion receiving chemotherapy (p = 0.08), radiation type (p = 0.22), CSI dose (p = 1), boost dose (p = 1), focal dose (0.08) or proportion who underwent surgery (0.57). Other patient characteristics and details of treatment can be reviewed in Table 1.

Table 2 reports the linear regression results of the 10 SNPs that showed the strongest association between allele (AA vs. other) and most recent IQ result, after adjusting for radiation type. Of the 10 SNPs reported, there was evidence that the association with most recent IQ score for 5 SNPS was not modified by radiation type. These 5 SNPs were located on 3 different genes (CYP2C9, XRCC1, and BRCA1) on 3 different chromosomes (chromosome 10, 19, and 17). BRCA1 on chromosome 17 had 3 different SNPs whose association with most recent IQ score was not modified by radiation type. Patients with the non-dominant allele in CYP2C9 were associated with a higher IQ compared to those who did not, after adjusting for radiation type (estimated least squares mean (95% CI): 93 (86,99) vs 74 (66,82), respectively). Patients with the dominant allele for XRCC1 on chromosome 19 had a higher IQ (estimated 90 (84,97) vs 74 (65,84) respectively) after adjusting for radiation type. Lastly, for all three SNPs in BRCA1 on chromosome 17, those with the dominant allele had a higher IQ compared to those without (estimated 93 (85,101) vs 78 (71,85)) after adjusting for radiation type. There were 5 SNPS in which there was evidence that the type of radiation received modified the association between allele and most recent IQ, and these results are presented in Table 2 where the Interaction Column is equal to “Y”.

Table 3 reports the results from linear regression for the 10 SNPs that showed the strongest association between allele and change in IQ, after adjusting for radiation type, first IQ, and years between first and second IQ measures. Of the 10 SNPs, there was evidence that for 5 SNPS the association with change in IQ score was not modified by radiation type, first IQ, or years between first and second IQ measures. These 5 SNPs laid on 4 different genes (WRN, NR3C1, ERCC4, RAD51L1) on 4 different chromosomes (chromosome 8, 5, 16, 14, respectively). Patients with the dominant SNP allele had a greater decrease in IQ for SNP rs12677942 on gene WRN (estimated decrease in IQ of −31 (95% CI: −46,−16) vs −1 (−6,3)), SNP rs2121152 on gene NR3C1 (−12(−18,−6) vs 2(−3,8)), SNP rs7185124 on ERCC4 (−38 (−59, −17) vs −2 (−7, 2)), SNP rs17106125 on gene RAD51L1 (−38 (−59, −17) vs −2 (−7, 2)), and SNP rs7712869 on gene NR3C1 (−21 (−34, −9) vs-1 (−6, 3)). There were also 5 SNPS in which there was evidence that the type of radiation received modified the association between allele and change in IQ, and these results are presented in Table 3 where the Interaction Column is equal to “Y”.

## Discussion

This study suggests the SNPs identified above could be an important tool to assess adverse neurocognitive outcomes in pediatric brain tumor survivors. Genetic polymorphisms are increasingly studied as possible predictors of treatment toxicity. Methotrexate, which is a folate antagonist, is an important component of leukemia therapy and is a well-described cause of neurocognitive toxicity [30]. In a study of 72 pediatric ALL survivors, genetic polymorphisms in genes involved in folate metabolism were found to correlate with deficits in attention and processing speed . While there have been a limited number of studies evaluating SNPs as predictors for neuropsychological impairment [20, 22], those that have been done restrict analysis to four or fewer genes at a time and usually focused on genes associated with antioxidant enzymes. We are not aware of any studies assessing genetic polymorphisms in many DNA damage repair genes in pediatric brain tumor survivors. The patients in the two IQ groups were well matched in clinical demographics allowing for a more robust analysis of the selected genetic markers. Furthermore, even after adjusting for potential confounders, several SNPs showed significant differences in IQ changes.

Of the 5 most important SNPs in which the interaction with most recent IQ score was not modified by radiation type, the SNPs were found in the *CYP2C9, XRCC1,* and *BRCA1* gene. *CYP2C9* is a cytochrome P450 protein that catalyzes many reactions involved in drug metabolism and synthesis of cholesterol, steroids, and other lipids (www.genecards.org). XRCC1 is a gene involved in repair of ionizing radiation and alkylating agent induced DNA single-stand breaks (www.genecards.org). BRCA1 encodes a nuclear phosphoprotein that plays a role in maintenance of genomic stability and secondarily acts as a tumor suppressor(www.genecards.org).

Furthermore, the SNPS on 4 different genes WRN, NR3C1, ERCC4, RAD51L1 whose interaction with change in IQ score was not modified by type of radiation, first IQ score, or time between first and second IQ score also have unique roles in DNA damage control and regulation. WRN encodes for a DNA helicase involved in DNA repair, NR3C1 a glucocorticoid response gene transcription activator, ERCC1 in nucleotide excision repair, and RAD51L1 in homologous recombination and repair (www.genecards.org).

Among the SNPs whose interaction with IQ score (XRCC1 and BRCA1) or change in IQ score (RAD51L1) was not modified by radiation type are part of the RAD51 pathway. RAD51 is the central recombinase protein involved in HR that is vital in the error free repair of DSBs within mammalian cells. Errors in precise HR have been shown to result in chromosomal abnormalities, immunodeficiency, neurodegeneration and cancer susceptibility.

Recruitment of RAD51 to the sites for repair depends on proper functioning of the RAD51 paralogs which include RAD51B, RAD51C, RAD51D, XRCC1, XRCC2 and XRCC3 [33]. Mutations in these paralogs have been shown to specifically attenuate RAD51 focus formation in response to irradiation and lead to the development of spontaneous chromosomal abnormalities [34]. Identification of multiple SNPs within this pathway may indicate a particularly important contribution of this pathway to long-term neurocognitive outcomes in this patient population. *RAD51, RAD52, BRCA1* and *BRCA2* genes have also been described as RAD associated factors important in RAD homologous recombination [31]. Furthermore, work by Berger et al, concluded that poor neurocognitive outcomes in children with pediatric brain tumors who underwent irradiation is likely associated with alterations in the RAD51 homologous recombination pathway {Berger, 2022 #1237}.

Early recognition of patients at highest risk for cognitive deficits due to therapy is important on many levels. It may be possible to identify patients who should be considered for reductions in certain therapies to reduce the risk of neurocognitive side effects, without reducing their long-term survival rates. For example, the Children’s Oncology Group standard risk medulloblastoma trial (COG ACNS0331, NCI clinical trials identifier NCT00085735) studied the reduction of radiation to the craniospinal axis to determine if radiation can be safely dose reduced to minimize neurocognitive late effects while maintaining high survival rates. Unfortunately, this study found that a reduced CSI dose led to an unacceptable increase in event rates and decreased survival, although it was possible to utilize smaller boost volumes in the posterior fossa [32]. If children at high risk of cognitive deficits but low risk of relapse could be identified at diagnosis, these children could potentially be preferentially included in dose-reduced treatment arms.

For children where reduction of radiation therapy would result in an unacceptable increase in treatment failure, early cognitive intervention may be important. Research has demonstrated that education interventions may help with improving long-term fluid intelligence and therefore overall cognitive performance. One study found that a short, computerized training session with 4-year-old subjects was able to improve their working memory, indicating early intervention might ameliorate school delays [33]. Another study evaluated the use of a computer training program to improve the working memory and reduce learning deficits of children born at extremely low birth weight (ELBW). Former ELBW adolescents who underwent a 5-week intervention program improved both trained and non-trained working memory. More importantly, those with an IQ <80 showed significant benefit, which was stable for at least 6 months after the training periods ended [34]. Cognitive rehabilitation has already been shown to improve fatigue, independence in activities of daily living, and overall cognitive function in pediatric cancer patients on therapy [35]. The Brainfit study is ongoing and looking specifically at cognitive and physical training as a potential treatment to improve neurocognitive outcomes in pediatric cancer survivors [36]. These studies highlight the importance of identifying children at risk for adverse neurocognitive outcomes, as there may be successful treatment strategies and potential interventions available to improve long-term educational and cognitive functioning.

These results support the hypothesis that somatic genetic variants in DNA repair genes may correlate with lower IQ scores in children treated for brain tumors and necessitate validation for potential use in clinical care and treatment planning.

## Figures and Tables

**Table 1:** Demographics and Clinical Characteristics

Characteristic	N	IQ<90 (n=26)	IQ>90 (n=20)	P-val
Time from First Treatment to Most Recent IQ^†^ *years*	42	5.1 (2.7, 7.6)	3.9 (2.7, 4.6)	0.13
Age at First Treatment ^†^ *years*	42	6.2 (4.3, 8.5)	6.2 (4, 11.2)	0.71
Most Recent IQ	46	73±14.6	101.4±11.4	<0.0001
Gender	46			0.99
Female		9 (35%)	6 (30%)	
Male		17 (65%)	14 (70%)	
Relapse	46			1
No		15 (58%)	11 (55%)	
Yes		11 (42%)	9 (45%)	
Diagnosis*	46			0.09
Craniopharyngioma		1 (4%)	3 (15%)	
EPN		2 (8%)	5 (25%)	
LGG		4 (15%)	2 (10%)	
MED		14 (54%)	4 (20%)	
NGGT		0 (0%)	1 (5%)	
Other		5 (19%)	5 (25%)	
Tumor Location*	46			0.02
4th ventricle		2 (8%)	0 (0%)	
Other		0 (0%)	1 (5%)	
Parietal		0 (0%)	2 (10%)	
Pineal		0 (0%)	2 (10%)	
Pituitary		0 (0%)	1 (5%)	
Posterior fossa		15 (58%)	9 (45%)	
Suprasellar/Hypothalamic		3 (12%)	5 (25%)	
Temporal		2 (8%)	0 (0%)	
Thalamus		4 (15%)	0 (0%)	
Chemotherapy*	46			0.08
No		3 (12%)	7 (35%)	
Yes		23 (88%)	13 (65%)	
Radiation Type	46			0.22
CSI and boost		15 (58%)	7 (35%)	
focal		11 (42%)	13 (65%)	
CSI Dose, categorical*	22			1
<=24 GY		11 (73%)	5 (71%)	
36 GY		4 (27%)	2 (29%)	
Boost Dose, categorical*	22			1
<20 GY		4 (27%)	2 (29%)	
>=30 GY		11 (73%)	5 (71 %)	
Focal dose	24	57.3±3.2	53.3±6.9	0.08
Surgery*	46			0.57
biopsy		4 (15%)	4 (20%)	
GTR		6 (23%)	7 (35%)	
STR		16 (62%)	9 (45%)	

† Skewed outcome: median (interquartile range) and Wilcoxon rank sum test

* Fisher’s Exact Test (due to small cell counts)

**Table 2:** The association between SNP allele (AA vs other) and most recent IQ scores between low (<90) and high (>90) IQ groups.

						Least square mean (95% CI) of recent IQ value			
SNP	Gene Name	Chromosome	pval	N	Interaction?	CSI, AA	Focal, AA	CSI, non- AA	Focal, non- AA
rs2107465	NBN	8	0.00052	43	Y	41 (17, 64)	110 (86, 133)	84 (77, 92)	88 (81, 95)
rs12772675	CYP2C9	10	0.00056	44	N	74 (66, 82)	74 (66, 82)	93 (86, 99)	93 (86, 99)
rs2735385	NBN	8	0.00082	43	Y	40 (17, 64)	103 (83, 122)	84 (77, 92)	88 (81, 95)
rs389480	BLM	15	0.00276	43	Y	91 (79, 103)	78 (66, 91)	74 (65, 84)	96 (87, 105)
rs3213403	XRCC1	19	0.00609	44	N	90 (84, 97)	90 (84, 97)	74 (65, 84)	74 (65, 84)
rs1374001	NR3C1	5	0.00621	43	Y	77 (68, 85)	94 (86, 103)	97 (79, 115)	78 (63, 92)
rs8110090	TGFB1	19	0.00696	43	Y	78 (70, 86)	94 (86, 102)	96 (75, 116)	70 (52, 88)
rs16940	BRCA1	17	0.00699	44	N	93 (85, 101)	93 (85, 101)	78 (71, 85)	78 (71, 85)
rs16942	BRCA1	17	0.00699	44	N	93 (85, 101)	93 (85, 101)	78 (71, 85)	78 (71, 85)
rs1060915	BRCA1	17	0.00699	44	N	93 (85, 101)	93 (85, 101)	78 (71, 85)	78 (71, 85)

**Table 3:** The association between SNP allele (AA vs other) and change in IQ scores between low (<90) and high (>90) IQ groups.

						Least square mean (95% CI) of change in IQ*			
SNP	Gene Name	Chromosome	pval	N	Interaction?	CSI, AA	Focal, AA	CSI, non- AA	Focal, non- AA
rs9514823	LIG4	13	0.00042	19	Y	−4 (−12, 5)	−24 (−37, −11)	−11 (−18, −5)	5 (−1, 11)
rs12677942	WRN	8	0.00076	20	N	−31 (−46, −16)	−31 (−46, −16)	−1 (−6, 3)	−1 (−6, 3)
rs2121152	NR3C1	5	0.00210	20	N	−12 (−18, −6)	−12 (−18, −6)	2 (−3, 8)	2 (−3, 8)
rs7185124	ERCC4	16	0.00294	20	N	−38 (−59, −17)	−38 (−59, −17)	−2	−2 (−7, 2)
rs17106125	RAD51L1	14	0.00294	20	N	−38 (−59, −17)	−38 (−59, −17)	−2	−2 (−7, 2)
rs7712869	NR3C1	5	0.00555	20	N	−21 (−34, −9)	−21 (−34, −9)	−1 (−6, 3)	−1 (−6, 3)
rs799917	BRCA1	17	0.00592	19	Y	−1 (−15, 14)	−20 (−36, −5)	−10 (−17, −3)	5 (−2, 11)
rs2347869	ESR1	6	0.00652	19	Y	−19 (−34, −4)	9 (0, 18)	−6 (−13, 1)	−8 (−17, 1)
rs726281	ESR1	6	0.00665	19	Y	−16 (−28, −4)	9 (0, 18)	−6 (−13, 1)	−8 (−17, 1)
rs330792	MSH6	2	0.00728	19	Y	−10 (−17, −2)	3 (−3, 10)	−6 (−18, 6)	−32 (−54, −11)

* Change in IQ = IQ_1 – IQ_2 (most recent – second most recent). So a positive change = increase in IQ over time, negative change = decrease in IQ over time.
